# Why one can expect large rectification in molecular junctions based on alkane monothiols and why rectification is so modest†
†Electronic supplementary information (ESI) available: Experimental and theoretical details, supplementary figures. See DOI: 10.1039/c8sc00938d


**DOI:** 10.1039/c8sc00938d

**Published:** 2018-04-09

**Authors:** Zuoti Xie, Ioan Bâldea, C. Daniel Frisbie

**Affiliations:** a Department of Chemical Engineering and Materials Science , Department of Chemistry , University of Minnesota , Minneapolis , Minnesota 55455 , USA . Email: frisbie@umn.edu; b Theoretische Chemie , Universität Heidelberg , INF 229 , D-69120 Heidelberg , Germany . Email: ioan.baldea@pci.uni-heidelberg.de

## Abstract

Many attempts to obtain high current rectification ratios (RRs) in molecular electronics are triggered by a potentiometer rule argument, which predicts that a strongly asymmetric location of the dominant molecular orbital yields large RR-values. Invoking this argument, molecular junctions based on alkane monothiols (CnT) can be expected to exhibit high RRs; the HOMO of these molecules is localized on the thiol terminal group bonded to one electrode. The extensive current–voltage (*I*–*V*) results for CP-AFM (conducting probe atomic force microscope) CnT junctions of various molecular lengths (*n* = 7, 8, 9, 10, and 12) and different metallic contacts (Ag, Au, and Pt) are consistent with conduction dominated by the HOMO, but the measured RR ∼ 1.5 is much smaller than that predicted by the potentiometer rule framework. Further, the linear shift in the HOMO position with applied bias, *γ*, which gives rise to rectification, is also smaller than expected, and critically, *γ* has the opposite sign from potentiometer rule predictions. Companion *ab initio* OVGF (outer valence Green's function) quantum chemical calculations provide important insight. Namely, a linear Stark shift *γ*_m_ is calculated for the HOMO of CnT molecules for electric field strengths (10^6^–10^7^ V cm^–1^) typical of molecular junctions, and the sign of *γ*_m_ matches the sign of the experimental *γ* for junctions derived from transport measurements, suggesting that the Stark effect plays an important role. However, the magnitude of the measured *γ* is only 10–15% of the computed value *γ*_m_. We propose that this implies that the contacts are far from optimal; they substantially screen the effect of the applied bias, possibly *via* molecule–electrode interface states. We predict that, with optimized contacts, the rectification ratios in CnT-based junctions can reach reasonably high values (RR ≈ 500). We believe that Stark shifts and limited current rectification due to non-ideal contacts discussed here for the specific case of alkane monothiol junctions are issues of general interest for molecular electronics that deserve further consideration.

## Abstract 2

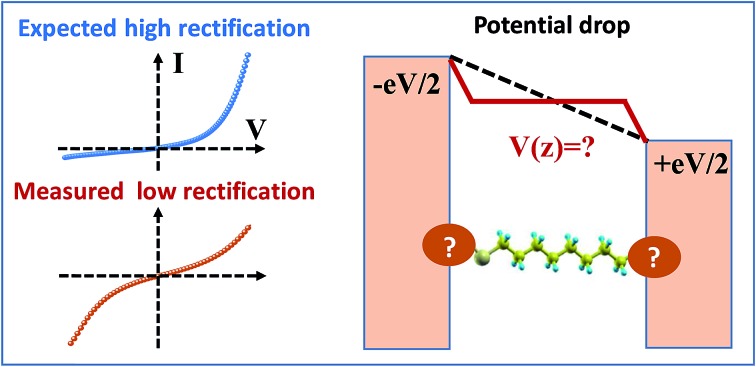
The Stark effect plays a key role in understanding why, against expectation, alkane thiols are not high-performance molecular rectifiers.

## Introduction

To become part of nanotechnology, molecular electronics should be able to fabricate molecular devices that supersede or at least undertake the basic functions of circuit components employed nowadays in semiconductor-based microelectronics.^1^–^15^ Rectification represents such an outstanding function, which has attracted the attention of the community since its inception.^16^ In molecular junctions where charge transport is dominated by a single molecular orbital (MO) one may intuitively expect that rectification is directly related to the bias-driven shift of the MO energy^17^–^20^ in principle determined by the local electric potential *φ*, which varies linearly across the junction (“potentiometer rule”) in the absence of screening.^19^–^22^ Guided by this potentiometer rule (schematically presented in Fig. S1†), tuning rectification by varying the position (*n*) of a ferrocene (Fc) unit (and thence the position of the dominant MO) within the alkyl chain of molecular junctions (S(CH_2_)_*n*_FcC_13–*n*_) has indeed been demonstrated.^14^,^19^

Although rectification ratios (RRs) comparable to the largest reported RR-values achieved recently (up to 10^5^) are not reached,^23^ ratios up to RR ∼ 100 obtained this way are significantly larger than those in many other cases (see ref. 24 for a review), making this ‘asymmetry approach’ to improve molecular rectification worthy of consideration. In view of the potentiometer rule, higher RR values can be expected in molecular junctions having the dominant MO very close to one electrode. In the example mentioned above, chemical synthesis ensured that the ferrocene unit could be precisely positioned, but it remained relatively distant from both molecular ends/electrodes.^23^,^25^ Molecular junctions based on alkane monothiols appear to be very attractive for fabricating high performance rectifiers because in these molecules, the dominant HOMO is well localized on the sulfur atom of the terminal thiol group bound to the substrate electrode (*cf.*Fig. 6A).

**Fig. 1:**
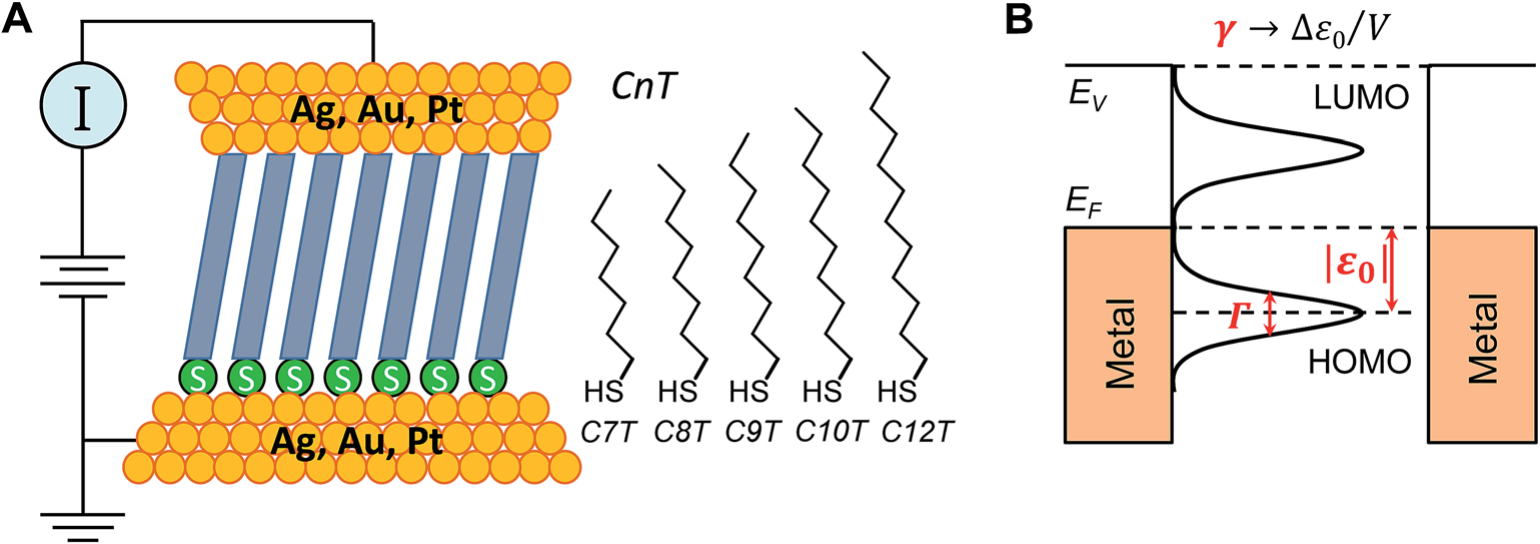
(A) Schematic representation of the CP-AFM setup. A metal-coated (Ag, Au, Pt) AFM tip is brought into contact with an SAM of alkane monothiols (CnT, *n* = 7, 8, 9, 10, 12) of various lengths on a metal coated substrate. (B) Typical junction electronic structure with key parameters: energy offset |*ε*_0_| (Fermi level relative to the HOMO level), molecule–electrode coupling strength *Γ*, and Stark effect strength *γ*.

In this work, 15 distinct molecular junctions were fabricated by using five alkane monothiols of different lengths (CnT, *n* = 7, 8, 9, 10, 12; examples in Fig. S2 of the (ESI†)) contacted by three different metallic (Ag, Au, and Pt) electrodes within the conducting probe atomic force microscope (CP-AFM) platform, see Fig. 1, which we introduced^26^ and employed in a variety of earlier molecular electronics studies.^27^–^33^ In view of our present focus, we only present results directly related to understanding the limitations of rectification, namely transport properties of CnT junctions in linear and nonlinear (transition voltage *V*_t_)^34^,^35^ bias ranges.

Nanojunctions based on alkanethiols are among the most investigated systems in molecular electronics.^26^,^36^–^51^ Therefore, it is important to mention the three new elements that distinguish the present study from earlier ones.

First, we employed analytical formulas^18^ deduced theoretically within a simple compact analytical model to interpret the transport measurements reported here. This model allows us to rationalize the whole body of asymmetric *I*–*V* transport data in terms of three parameters having a clear physical meaning. Building on the two-parameter model (HOMO offset |*ε*_0_| and coupling *Γ*) utilized earlier for symmetric junctions,^32^,^33^,^51^,^52^ we employ an extra (third) parameter, namely the Stark effect strength *γ* (Fig. 1B). Quantifying the bias-driven shift of the dominant (HOMO) transport energy level, the parameter *γ* is able to account for the asymmetry of the measured *I*–*V* curves upon bias polarity reversal and, thence, to characterize rectification.

Second, by performing accurate *ab initio* quantum chemical calculations based on the outer valence Green's function (OVGF) method,^53^,^54^ we are able to estimate the bias-driven HOMO energy shift and obtain a theoretical estimate *γ* → *γ*_m_ of the Stark effect strength for isolated molecules.

Third, we demonstrate that the CP-AFM CnT junctions diverge from the predictions of the potentiometer framework in two important respects: (1) the sign of the bias-induced HOMO energy shift is opposite to the value one would predict given the spatial location of the HOMO; (2) the junctions exhibit weak rectification (RR ∼ 1.5–2). The first observation is explained in terms of the above mentioned Stark effect. In fact, the potentiometer rule does not hold in CnT systems; instead electric fields in the junction interact with the HOMO to produce Stark shifts *γ* with a sign that matches the sign of the *γ* values extracted from transport measurements. Yet the magnitude of the measured *γ* for junctions is only 10–15% of the computed value *γ*_m_ for isolated molecules. Contact effects are likely the cause that the applied bias (and hence electric field) felt by the molecules in the junction is small, giving rise to a smaller *γ*. The lower value of *γ* is in turn responsible for the low RR (second observation).

Notwithstanding the very modest measured RR-values, the analysis emerging from the present joint experimental–theoretical study provides significant insight into the understanding of molecular rectification going beyond the specific case of CnT. The difference between the values *γ* and *γ*_m_ obtained as outlined above, which is substantial, is likely related to phenomena occurring at molecule–electrode interfaces and demonstrates that the latter are very important for rectification. This finding provides a fresh look at the problem of molecular current rectification and highlights the surprisingly important roles that the Stark effect and contacts (possibly interface states) have in this context.

## Results and discussion

### Basic working equations

To interpret transport measurements on our CP-AFM junctions based on CnT, we employ below the basic *I*–*V* equation deduced in ref. 18 by assuming transport by tunneling determined by a single dominant energy level (MO), which is possibly shifted by applied bias (*cf.*eqn (2)), and a Lorentzian-shaped transmission1
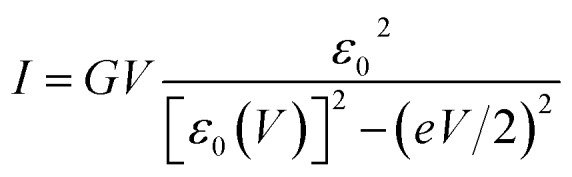
Here *ε*_0_ = *E*_MO_ – *E*_F_ represents the MO energy offset relative to the Fermi level characterizing the unbiased junction. In general, the MO energy *E*_MO_(*V*) in a biased junction (*V* ≠ 0) is shifted with respect to its position in the absence of bias *E*_MO_ ≡ *E*_MO_(*V* = 0) by a quantity proportional to *V*^17^,^18^

2 *E*_MO_(*V*) = *E*_MO_ + *γeV*, *ε*_0_(*V*) ≡ *E*_MO_(*V*) – *E*_F_ = *ε*_0_ + *γeV*

In view of possible screening effects and arguments presented later, rather than the voltage division (potentiometric) factor,^17^,^18^ the dimensionless quantity *γ* in eqn (2) should be more properly referred to as the Stark effect strength.^22^

A discussion of the sign of *γ* is in order at this point. We define the bias polarity such that a positive bias (*V* > 0) corresponds to the tip having a higher electric potential than that of the substrate (“tip positive, substrate negative”). In real CnT junctions, methyl terminal groups are coupled to the tip while thiol groups are coupled to the substrate. Adopting this convention, in the quantum chemical calculations presented below, a positive bias corresponds to an electric field (which is an input parameter in our calculations with GAUSSIAN 09, see ESI†) oriented from methyl to thiol. According to eqn (2), a bias *V* of a given polarity shifts the MO energy upwards or downwards depending on the sign of *γ*. Equivalently, the sign of both *V* and *γ* determine the direction of the MO shift. For negative *γ* (*γ* < 0, as turns out to be the case of the presently investigated CnT-based junctions), a positive bias (*V* > 0, *γV* < 0) causes a downward shift of the MO energy, whereas a negative bias (*V* < 0, *γV* > 0) yields an upward shift of the MO energy. This means that, in cases where *γ* < 0 and conduction is dominated by the HOMO (as is the case of CnT-based junctions, see below), a positive bias takes the HOMO away from the Fermi energy, thereby reducing the current, while a negative bias brings the HOMO closer to the Fermi level, thereby enhancing the current. Therefore, for *γ* < 0 and HOMO conduction, the HOMO level tracks the tip (*cf.*, Fig. 4) and currents are higher for negative biases than for positive biases. In contrast, in cases where *γ* < 0 and conduction is mediated by the LUMO, the energy shift would be decreased (LUMO closer to the Fermi level) for *V* > 0 and increased (LUMO more distant from the Fermi level) for *V* < 0; this would result in higher currents at positive biases than at negative biases.

Returning to the single level model, the zero-bias conductance *G* = 1/*R* of the CP-AFM junction can be expressed as follows:3
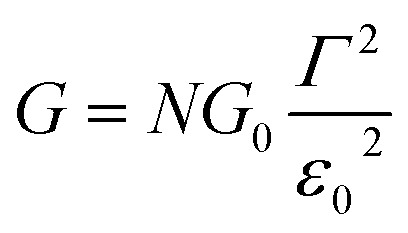


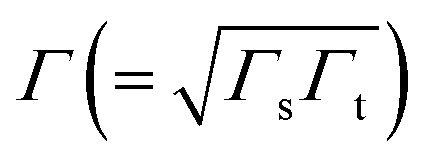
 being the geometrical average of the couplings *Γ*_s_ and *Γ*_t_ between the MO and the substrate (s) and tip (t) electrodes, *G*_0_ = 2*e*^2^/*h* is the quantum conductance, and *N* is the number of molecules contributing to the transport through the CP-AFM junction.

In the case of molecular junctions with asymmetric *I*–*V* characteristics, the quantity *V*^2^/|*I*|^51^,^55^ exhibits two maxima asymmetrically located at biases of opposite polarities (*V*_t+_ > 0, *V*_t–_ = –|*V*_t–_| < 0) and different magnitudes (*V*_t+_ ≠ –*V*_t–_) defining two transition voltages. They can be used to estimate the magnitude of the energy offset of the occupied level (*ε*_0_ = –|*ε*_0_| = *E*_HOMO_ – *E*_F_ < 0) that dominates the charge transport and the Stark effect strength *γ* as follows^18^,^32^,^55^
4
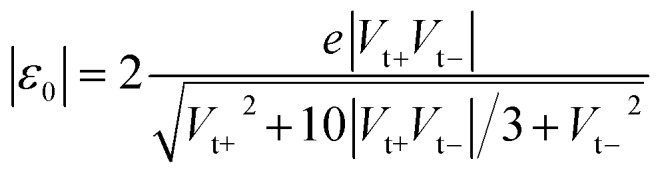

5
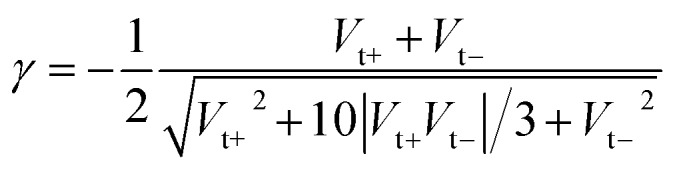



### Low bias resistance *R*

The measured low bias *R*-data presented in Table 1, which shows the main results for our CP-AFM junctions, reveal two important aspects. First, *R* exponentially increases with the molecular size *n* (*cf.* Fig. S3A†), in accord with eqn (S5),† wherein the dependencies on size (*n*) and contact (*R*_c_) are disentangled. This exponential dependence is well known^36^,^40^–^46^ and represents a clear indication of transport *via* off-resonant tunneling. Second, *R* (and *R*_c_) dramatically decreases as the electrode work function *Φ* increases (*cf.* Fig. S3B†). Given the fact just noted that the transport occurs by tunneling and the fact that transport data are well described within a single level picture (see the section “Self-consistency check: simulation of full *I*–*V* curves using the single level” below), this dependence on *Φ* represents unambiguous evidence that charge transport in CnT junctions is mediated by an occupied level.

**Table 1:** Summary of the main results for CnT CP-AFM junctions. Low bias resistance *R* and contact resistance *R*_c_ of the junctions in Ω, average attenuation factor *β* (per carbon), transition voltages *V*_t±_ in V, energy offset |*ε*_0_| in eV, Stark effect strength *γ* (dimensionless), and coupling *Γ* in meV. The number of the molecules *N* = 70 (*cf.* ESI) was used for calculating *Γ* as described in the ESI and depicted in Fig. S6

Electrodes	Quantity	C7T	C8T	C9T	C10T	C12T
Ag–Ag, *β* = 1.16, *R*_c_ = 3.5 × 10^4^	*R*	6.89 × 10^7^	4.20 × 10^8^	9.78 × 10^8^	3.86 × 10^9^	3.65 × 10^10^
	*V*_t–_	1.26	1.23	1.22	1.20	1.21
	*V*_t+_	1.46	1.39	1.42	1.44	1.44
	|*ε*_0_|	1.17	1.13	1.14	1.13	1.14
	*γ*	–0.032 ± 0.008	–0.026 ± 0.007	–0.033 ± 0.007	–0.039 ± 0.01	–0.038 ± 0.009
	*Γ*	1.92	0.75	0.49	0.25	0.08
Au–Au, *β* = 1.19, *R*_c_ = 1.5 × 10^3^	*R*	6.17 × 10^6^	3.14 × 10^7^	8.47 × 10^7^	3.07 × 10^8^	2.85 × 10^9^
	*V*_t–_	1.06	1.05	1.00	1.02	1.01
	*V*_t+_	1.28	1.31	1.22	1.25	1.29
	|*ε*_0_|	1.01	1.01	0.95	0.97	0.98
	*γ*	–0.041 ± 0.011	–0.048 ± 0.011	–0.043 ± 0.015	–0.044 ± 0.013	–0.053 ± 0.014
	*Γ*	5.50	2.45	1.41	0.75	0.25
Pt–Pt, *β* = 1.16, *R*_c_ = 3.1 × 10^2^	*R*	8.11 × 10^5^	4.59 × 10^6^	1.17 × 10^7^	4.02 × 10^7^	4.10 × 10^8^
	*V*_t–_	0.94	0.93	0.91	0.86	0.88
	*V*_t+_	1.18	1.19	1.15	1.09	1.15
	|*ε*_0_|	0.91	0.91	0.88	0.83	0.87
	*γ*	–0.049 ± 0.008	–0.053 ± 0.01	–0.051 ± 0.009	–0.051 ± 0.01	–0.058 ± 0.011
	*Γ*	13.68	5.74	3.50	1.79	0.58

### General *I*–*V* behavior in a nonlinear bias range and transition voltage

**Fig. 2:**
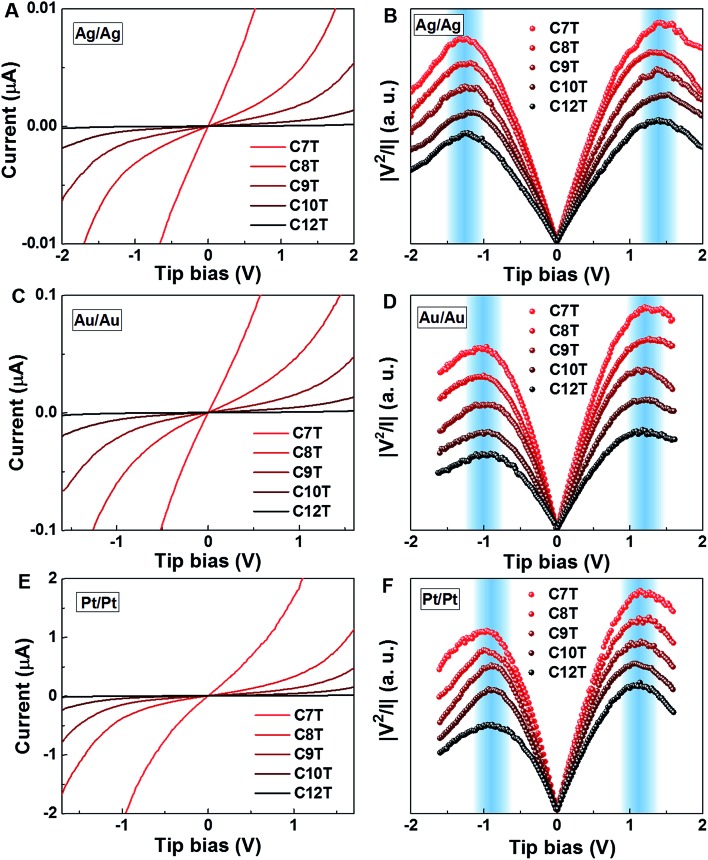
Representative averaged *I*–*V* traces and transition voltage spectra of alkane monothiols CnT (*n* = 7, 8, 9, 10, 12) embedded in (A, B) Ag/Ag, (C, D) Au/Au, and (E, F) Pt/Pt CP-AFM junctions.

Fig. 2A, C and 2E display representative full *I*–*V* characteristics of CP-AFM junctions based on CnT (*n* = 7, 8, 9, 10, 12) with various metallic electrodes (tip, substrate = Ag, Au or Pt). The semilogarithmic *I*–*V* traces are shown in Fig. S4.† For a given bias and electrode type, currents decrease exponentially with the length. For a given molecular species (*i.e.*, fixed *n*) and bias, currents increase as the electrode work function increases. Again, these two features indicate hole transport *via* tunneling mediated by an occupied level.

For a more comprehensive examination of transport properties as in our previous study on alkane dithiols,^36^ we investigated the full *I*–*V* characteristics over the interval ±1.6 V (±2.0 V for Ag/Ag junctions), recasting them as curves of *V*^2^/|*I*| *versus V* (Fig. 2B, D and F). This type of plot exhibits two peaks, which define the (transition) bias (*V* = *V*_t_) for either bias polarity (*V*_t_ → *V*_t±_) where the differential conductance is two times larger than the nominal (pseudo-ohmic) conductance 
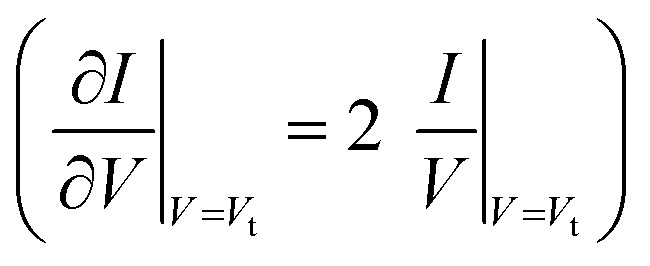
. This approach is an alternative reformulation of transition voltage spectroscopy (TVS),^34^ as it can be shown that the voltage at the peak maximum and the transition voltage *V*_t_ (defined as the bias at the minimum of the Fowler–Nordheim plot) are mathematically identical.^55^ In contrast to the symmetric *I*–*V* curves of alkane dithiol junctions,^33^ the *I*–*V* curves of the present CnT exhibit a significant asymmetry [*I*(–*V*) ≠ –*I*(*V*)]. Accordingly, the magnitude of the transition voltages at positive (*V*_t+_ > 0) and negative (*V*_t–_ < 0) biases for CnT differs (*cf.*Table 1). Table 1 and Fig. S5A† show that the average value at positive bias polarity (*i.e.* the tip in contact with the methyl end is positively polarized with respect to the substrate in contact with the thiol anchoring group) *V*_t+_ is ∼0.2 V larger than the magnitude of the average value |*V*_t–_| at negative bias polarity (*i.e.* the tip in contact with the methyl ends is negatively polarized with respect to the substrate in contact with the thiol anchoring group).

**Fig. 3:**
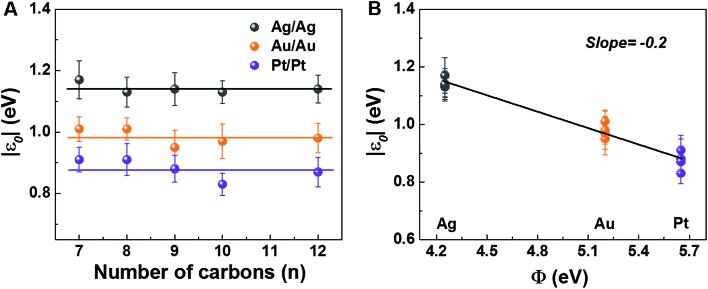
HOMO energy offset relative to the Fermi level of electrodes for M–CnT–M junctions (M = Ag, Au, Pt; *n* = 7, 8, 9, 10, 12) as a function of (A) molecular length and (B) bare electrode work function. The lines represent linear fits.

The experimental values of *V*_t±_ and eqn (4) allow us to determine the energy offset of the dominant level |*ε*_0_| (see ESI† for details). For a given metal contact, our data show no significant length dependence of *V*_t_ (Fig. S5A†), in agreement with previously reported *V*_t_ values of alkane monothiols on Au electrodes.^56^*Via*eqn (4), this results in *n*-independent |*ε*_0_|-values (Fig. 3A). On the other hand, the absolute values of *V*_t±_ decrease with increasing the work function of the electrodes (see Fig. S5B†). This yields |*ε*_0_|-values decreasing with increasing *Φ* (see Fig. 3B), which is consistent with the fact that charge transport is mediated by an occupied level.

#### Stark effect strength *γ*

 Eqn (5) allows us to determine the Stark strength *γ*. Notice that the only quantity needed is the ratio |*V*_t+_/*V*_t–_|. So, the fact that |*V*_t+_/*V*_t–_| does not depend on *n* (*cf.* Fig. S5C†) implies that *γ* is also *n*-independent, which is a significant aspect for the discussion that follows. The average values and statistical deviations of *γ* are presented in Table 1.

**Fig. 4:**
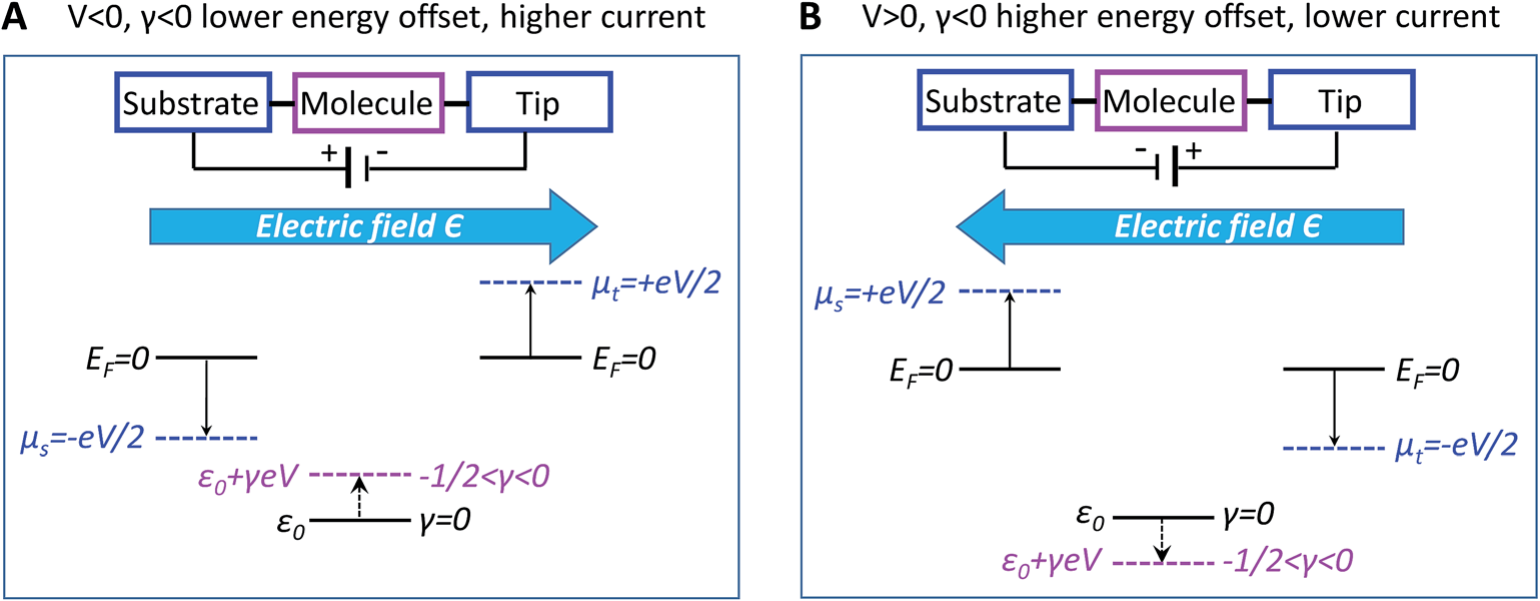
Energy diagram at (A) negative bias *V* ≡ *V*_t_ – *V*_s_ < 0 and (B) positive bias *V* ≡ *V*_t_ – *V*_s_ > 0. The negative value of *γ* of CnT junction yields |*ε*_0_(*V* < 0)| < |*ε*_0_(*V* > 0)|, and thus |*I*(*V* < 0)| > |*I*(*V* > 0)|. Note that the HOMO level, which is localized near the substrate, appears to track the tip potential, which is opposite to the predictions of the potentiometer framework. We claim that the HOMO shifts under bias are actually due to the Stark effect.

In spite of some statistical spread (see the *γ*-histograms of Fig. S7†), there is a clear indication that the sign of *γ* obtained in this way is negative. By virtue of eqn (2), this implies that an electric field pointing toward the methyl group (negatively polarized tip, *V* < 0) yields an upward shift of the dominant level energy while an electric field pointing toward the thiol group (positively polarized tip, *V* > 0) yields a downward level shift, as shown in Fig. 4A and B, respectively (see earlier discussion of the sign of *γ*).

As we will see below, corroborating the information on *γ* extracted from the measurements with the results of quantum chemical calculations yields important new insight into current rectification. We note for now that the negative value of *γ* contradicts predictions based on the potentiometer rule as the HOMO, localized on the thiol bonded to the substrate, could be expected to track the substrate potential, whereas the HOMO energy instead appears to track the tip potential (*γ* < 0 means the HOMO tracks the tip potential).

### Self-consistency check: simulation of full *I*–*V* curves using the single level model

**Fig. 5:**
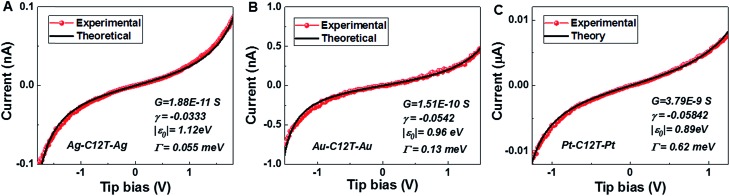
The good agreement between the individual experimental *I*–*V* curves (red) for C12T and those obtained theoretically *via*eqn (1) (black) is illustrated here for (A) Ag/Ag, (B) Au/Au and (C) Pt/Pt junctions. For each junction, the three parameters needed in eqn (1) – low bias conductance *G* (*1*/*R*), energy offset |*ε*_0_| and *γ* – are indicated in the legends.

With the model parameters |*ε*_0_|, *γ*, and *Γ* determined from the experimental *V*_t±_- and *G*-values *via*eqn (3)–(5) we are able to reproduce well the individual *I*–*V* traces measured for CnT junctions. Some representative *I*–*V* theoretical curves obtained *via*eqn (1) and (2) superimposed on the corresponding measured *I*–*V* traces in the bias range (|*V*| < 1.5|*ε*_0_|/*e*) where eqn (1) applies^18^ are presented in Fig. 5 and S8.† As visible there, similar to the cases of symmetric *I*–*V* curves (*γ* = 0, *V*_t+_ = –*V*_t–_ = *V*_t_),^32^,^33^ the agreement between the theory based on the single level model and experiment is also very good for the present case wherein the *I*–*V* curves are asymmetric (γ ≠ 0, *V*_t+_ ≠ –*V*_t–_). Noteworthy is the fact that, similar to the case of molecular junctions with symmetric *I*–*V* characteristics, to determine our model parameters, we need not fit the full *I*–*V* curves; we only fix these model parameters as described above. Of course, the fact that the *I*–*V* curves calculated in this way excellently reproduce the experimental curves represents a significant self-consistency check for the model based on the bias-driven shifted single level, thereby validating this model for the benchmark case of molecular (CnT) junctions with asymmetric *I*–*V* characteristics.

To end this part, the presently employed model provides us with a better understanding of the properties of the CP-AFM junctions investigated here than the Simmons model used earlier by our group^36^ and others^43^,^44^ for the same type of junctions.

### Important insight into charge transport aided by quantum chemical calculations

The foregoing discussion indicated that the overall increase in currents at both low and higher biases with increasing electrode work function observed in experiments is incompatible with conduction dominated by the LUMO.^57^ The *I*–*V* measurements presented above demonstrated that the charge transport in the present CP-AFM molecular junctions based on alkane monothiols can be explained by assuming that a single occupied level is dominant. On this basis, a LUMO-mediated conduction should be ruled out (for further arguments against LUMO conduction see the discussion related to Fig. S9 in the ESI†). So, a conduction dominated by the closest occupied MO to the metallic Fermi energy (which is the HOMO) appears to be the most plausible assumption. A series of results of *ab initio* calculations at the OVGF level of theory^53^,^54^ using 6-311++g(d,p) basis sets for all atoms will be presented below that reveal similar behaviors of the HOMO energies calculated for isolated CnT molecules and the energies of the occupied level found to dominate the charge transport in CnT-based junctions, thereby supporting the assumption of a HOMO-mediated conduction.

#### (i) *n*-Independence of the HOMO energy

Our OVGF-based calculations for isolated alkane monothiol molecules (CnT, *n* = 7, 8, 9, 10, 12) yielded values of the HOMO energy practically independent of the molecular size *n*. Because this behavior is similar to our recent report for alkane dithiol junctions^33^ as well as the length independent band gap of molecules possessing saturated hydrocarbon backbones,^58^–^60^ the results for HOMO energies in the CnT series are not shown here. Most importantly from the present standpoint, this behavior is similar to the *n*-independent values of |*ε*_0_| deduced from the transport measurements (*cf.*Table 1).

#### (ii) Linear dependence of the HOMO energy on *V*

**Fig. 6:**
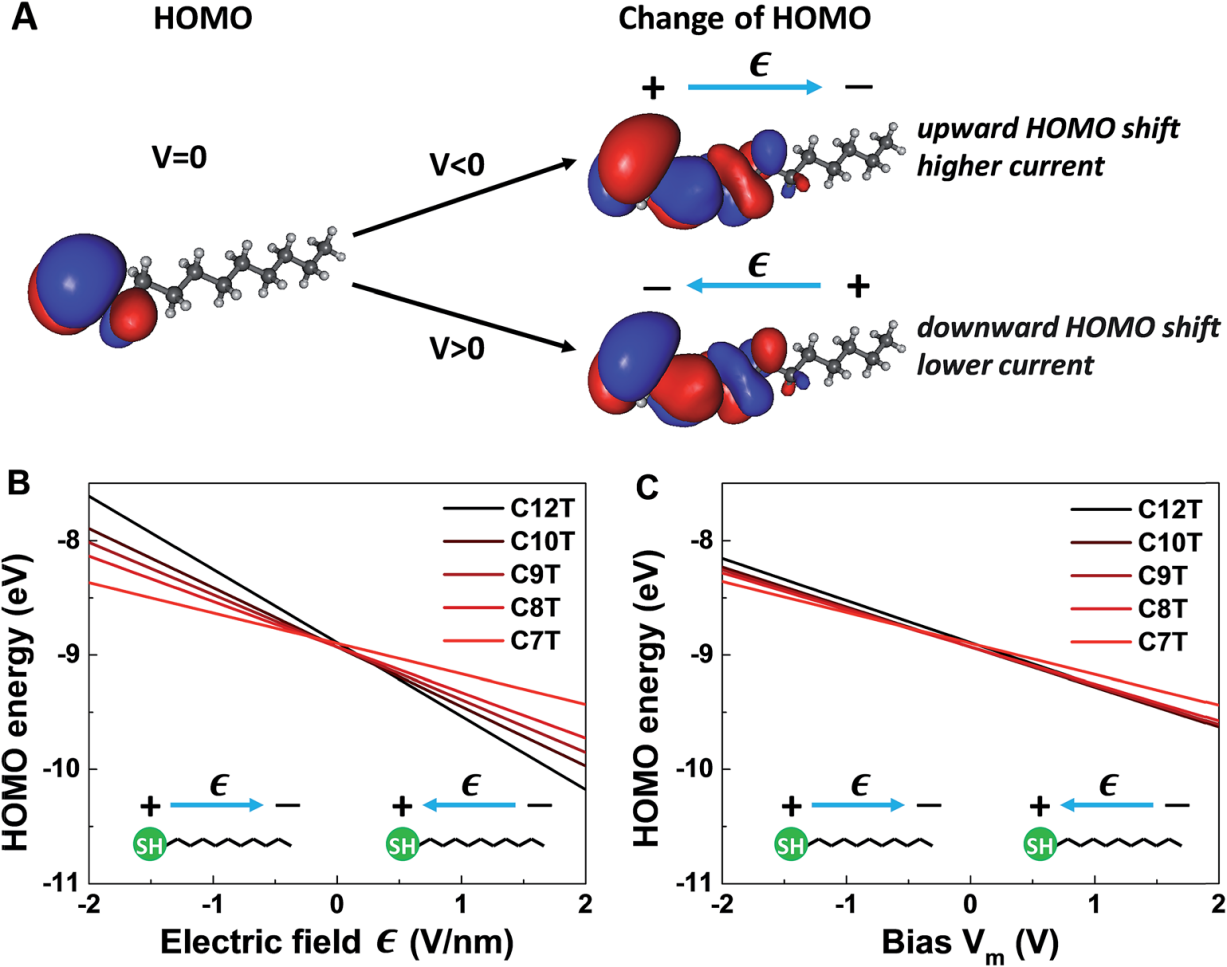
Results of quantum chemical OVGF computations for isolated molecules. (A) Schematic representation of the spatial distribution of the HOMO of C10T at zero bias and its changes under the different polarities. Notice that in panel A the picture in the left part shows the HOMO distribution *ρ*_HOMO_(*r*, *V* = 0) computed without bias, while the pictures in the right part show computed changes – which are very small – in the HOMO distributions *δρ*_HOMO_(*r*, *V*_m_) ≡ *ρ*_HOMO_(*r*, *V*_m_) – *ρ*_HOMO_(*r*, *V* = 0) brought about by biases |*V*_m_| ≈ 1.5 V depicted by using an isovalue representing ∼0.7% of the isovalue employed to depict the HOMO at zero bias (*i.e.*, in the left picture). (B) HOMO energies of isolated alkanethiol molecules CnT (*n* = 7, 8, 9, 10, 12) as a function of the applied electric field **Є**. (C) HOMO energies as function of bias *V*_m_ between the molecular ends. The slopes of the lines in (C) represent *γ*_m_ < 0. We note that OVGF calculations yield a linear dependence for biases much higher than the V-range of experimental interest shown here.

OVGF-calculations for isolated CnT molecules placed in an external electric field *Є* along the molecular axis yield HOMO energies that linearly depend on *Є* (see Fig. 6). This linear dependence on the *Є* of the OVGF-based HOMO energy values translates into a linear dependence on the bias *V* in the entire bias range of experimental interest (*cf.*Fig. 6C)

6 *E*_HOMO,m_(*V*) = *E*_HOMO,m_ + *γ*_m_*eV*_m_ = *E*_HOMO,m_ – |*γ*_m_|*eV*_m_

here, *V*_m_ ≡ *Єd*_HH_, where the molecular length *d*_HH_ is taken between the most distant hydrogen atoms of the CnT molecules estimated by geometry optimization at the DFT/B3LYP/6-311++g(d,p) level. The subscript m denotes the calculated results for isolated molecules.

It is worthwhile noting that an applied field/bias acts as a perturbation on the molecule that in principle may cause corrections to both the HOMO energy (eqn (6)) and HOMO spatial density. From the point of view of quantum mechanics, the HOMO energy is an eigenvalue, and the HOMO spatial distribution is expressed by *ρ*_HOMO_(*r*) ≡ |*Ψ*(*r*)|^2^, where its wave function *Ψ* is an eigenfunction. Quantum mechanics (*e.g.*, ref. 61) tells us that, if corrections to eigenvalues are of first order, corrections to eigenfunctions are negligible (first-order corrections to eigenvalues can be accurately calculated with eigenfunctions of the unperturbed system). The very weak impact of the applied bias on the HOMO spatial distribution is illustrated in Fig. 6A. The left panel of Fig. 6A depicts the HOMO spatial density *ρ*_HOMO_(*r*, *V* = 0) in the absence of bias generated with GABEDIT^62^ by using an isovalue of 1.3 × 10^–2^ a.u. (atomic units). The right panel visualizes changes in the HOMO distribution *ρ*_HOMO_(*r*, *V* ≠ 0) at biases close to the highest values used in experiment. If we depicted those HOMO distributions *ρ*_HOMO_(*r*, *V* = ±1.5 V), their very small differences from the left panel at *V* = 0 would be invisible within any reasonable drawing accuracy. Therefore, to give a flavor of these tiny changes, in the right panels of Fig. 6A we present changes in the HOMO density *δρ*_HOMO_(*r*, *V*) ≡ *ρ*_HOMO_(*r*, *V*) – *ρ*_HOMO_(*r*, *V* = 0) by using a much smaller isovalue of 9.4 × 10^–5^ a.u.

We noted above that the dependence of the HOMO energy (eqn (6)) is found to be linear (*i.e.*, of first order in *V*). This implies that an applied bias negligibly affects the HOMO wave function. In turn, this means that bias-driven changes to the HOMO spatial density are altogether negligible. For this reason, MO (HOMO or else) spatial distributions are not significantly altered by applied biases in all other cases where quantum chemical calculations yield a linear dependence on the *V* of the type expressed by eqn (6). In the same vein, improving rectification by a bias-driven enhancement of the asymmetry of MO distributions can hardly be expected.

The linear dependence of the HOMO energy expressed by eqn (6) is analogous to the linear behavior of the energy of the occupied level found to dominate the transport in CnT junctions expressed by eqn (2). Note also that the calculated linear dependence exhibited in Fig. 6 is a direct consequence of the strongly inhomogeneous spatial distribution of the HOMO (see below).

#### (iii) Sign of *γ*

Our OVGF-calculations indicated that an electric field directed toward the methyl (thiol) group raises (lowers) the HOMO energy (Fig. 6A). Noting that, both in eqn (2) and in eqn (6), a positive bias (*V* for junctions or *V*_m_ for isolated molecules) means an electric potential at the methyl (tip) end higher than the electric potential at the thiol (substrate) end, this result implies that the energies of the HOMO (for an isolated molecule) and of the occupied level that dominates the transport in a junction exhibit a similar qualitative behavior, namely a linear dependence on bias with a negative slope; *γ* of eqn (2) and *γ*_m_ of eqn (6) have the same negative sign.

#### (iv) *n*-Independent slope *γ*_m_

As visible in Fig. 6B, at a given field strength *Є*, longer molecular species exhibit larger HOMO energy shifts than shorter species. However, by recasting the dependence of the HOMO energy on the electric field *Є* of Fig. 6B (which is the usual manner of depicting the Stark effect^61^) as HOMO energy *versus* bias (*V*_m_ = *Єd*_HH_), we found that the straight line of *E*_HOMO_*vs. V*_m_ is practically independent of the molecular size *n* (*cf.*Fig. 6C). The weak spread in the straight lines corresponding to the various molecular species visible in Fig. 6C can be attributed to the unavoidable uncertainty in defining the lengths of molecules that are not strictly linear. The *n*-independence of the slope *γ*_m_ is similar to the behavior of *γ* deduced from our transport data; within errors, the values of *γ* are also independent of *n* (*cf.*Table 1). This *n*-independence suggests that, rather than the backbone length, it is the physics at the molecular ends that plays an essential role in the bias-driven level shift.

So, as anticipated, the results of quantum chemical calculations presented above support the assumption of a HOMO-mediated conduction in CnT junctions. They reveal a series of similarities between the HOMO energies and the values extracted from transport data of the energies of the single level found to dominate the charge transport, which indicates that it is plausible to ascribe the occupied dominant level to the HOMO. Out of these similarities, the fact that both *γ*_m_ and *γ* are negative is particularly noteworthy, as it is related to a counterintuitive behavior. According to common intuition, in a molecular junction under applied bias, the dominant energy level follows the “motion” of the Fermi level of the electrode to which it is strongly coupled. A CnT molecule has its HOMO localized on the thiol group (*cf.*Fig. 6A), very close to the substrate, on which it is chemisorbed. Therefore, one can expect that the HOMO energy will follow the substrate's Fermi energy and not the Fermi energy of the tip, on which the molecule is physisorbed. This behavior would correspond to *γ* > 0. This would be just opposite to the situation depicted in Fig. 4, which is the picture compatible with the transport measurements. This “intuitive” argument assumes that simple classical electrostatics dictates the behavior of the potential across the junction, smoothly interpolating between the substrate and the tip according to the potentiometer rule. The OVGF results shown here do not support this classical electrostatic description. They emphasize the fact that there is a quantum mechanical contribution to the MO-shifts driven by the electric field;^22^ rather than a voltage division (potentiometric) factor,^17^,^18^ *γ* of eqn (2) (and *γ*_m_ of eqn (6)) represents a Stark effect strength.^22^ Note that the linear dependence on *Є* (and on bias) is the direct consequence of the strongly inhomogeneous spatial distribution of the relevant level; the linear contribution in *Є* would vanish and the correction to the level energy would be proportional to *Є*^2^ if the spatial distribution was homogeneous, like in the case of the Stark effect for atoms.^61^

**Fig. 7:**
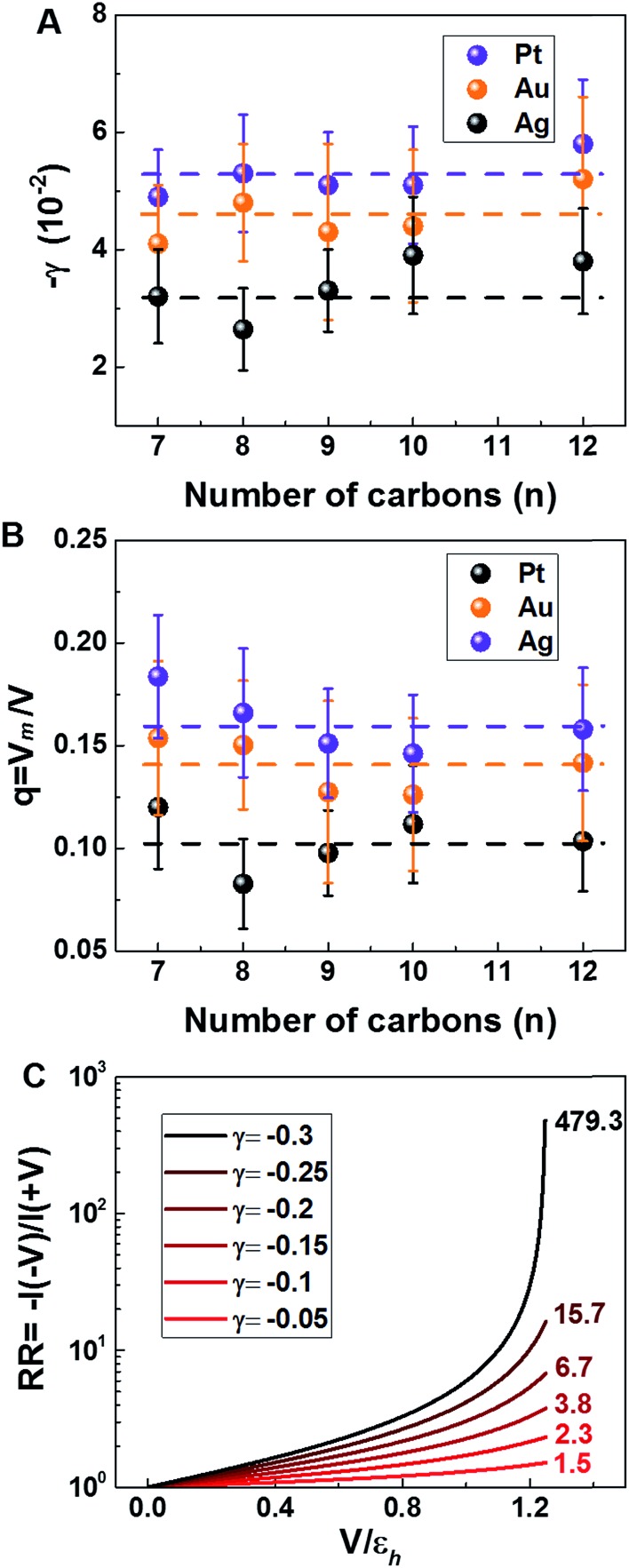
(A) Stark effect strength *γ* of M–CnT–M junctions. Within errors (standard deviations *δγ* are shown here as error bars), the experimental values of *γ* are independent of the molecular size *n*. (values of *γ* and *δγ* are from Table 1). (B) The bias *V*_m_ felt by the HOMO only represents a small fraction *q* (depicted in this panel) from the total bias *V* applied between the tip and the substrate. Here *q* = *V*_m_/*V* = *γ*/*γ*_m_ where *γ* comes from panel (a) (*i.e.*, experiment) and *γ*_m_ is the result of calculations (see Fig. 6C). (C) Computed rectification ratio RR ≡ –*I*(–*V*)/*I*(*V*) for several values of the Stark effect strength *γ* ranging from the experimental value (*γ* ≈ –0.05, *cf.*Table 1) and the theoretical OVGF value (*γ* ≈ –0.3).

More quantitatively, one should note that *γ* of eqn (2) describes the bias-driven shift of the HOMO energy of a molecule embedded in a CP-AFM junction, while *γ*_m_ of eqn (6) refers to the HOMO of an isolated molecule. If we identified the bias *V*_m_ with the experimental tip-substrate bias *V*, the slopes of Fig. 6C (*γ*_m_) would be almost one order of magnitude larger than the values of *γ* extracted from experiment (*cf.*Table 1, Fig. 7A). We interpret this result – and consider it as a key finding of this study – as evidence that the HOMO does not respond to the entire bias (*V*) applied on the junction; what the HOMO energy feels is only a small fraction *q* of the bias applied between the tip and the substrate:7
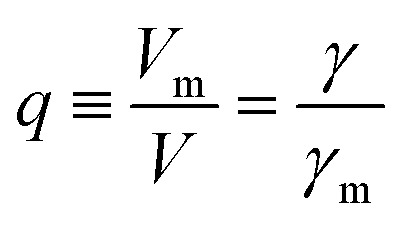



This ratio *q* is represented in Fig. 7B. As already noted, the values *q* ≈ 0.1–0.15 visible in Fig. 7B indicate that only a small amount of the applied bias (10–15%) is felt by the HOMO. This necessarily implies that, at least for the presently considered molecular junctions based on alkanethiols, the contacts are far from optimal; they substantially mitigate the effect of the applied bias on the HOMO energy of the embedded molecules.

#### Rectification-related issues

As seen above, *I*–*V* data demonstrate that nanojunctions based on alkane monothiols act as weak molecular rectifiers: currents at positive biases *V* (>0, positive tip polarity) are lower than currents at negative biases: *I*(+*V*) < |*I*(–*V*)|. The rectification ratios RR ≡ –*I*(–*V*)/*I*(*V*) are in the range of 1.5–2 at 1.5 V for alkane monothiol junctions. According to eqn (3) this weak current rectification property traces back to the fact that the energy level offset |*ε*_0_(*V*)| at positive tip biases (*V* > 0) is larger than that at the same bias of opposite polarity (–*V* < 0); that is, |*ε*_0_(–*V* < 0)| < |*ε*_0_(*V* > 0)|, as depicted in Fig. 4.

Recently, various groups succeeded in fabricating molecular devices exhibiting rectification ratios (RRs) larger than 10^2^ or even 10^5^.^12^–^15^,^23^,^24^,^63^–^68^ In view of such achievements, it is hard to discuss “rectification” based on the very modest values RR ≈ 1.5 that characterize CnT junctions. However – and this is the important point we want to make here – these small RR-values are merely a consequence of the fact that the HOMO of CnT does not respond to the entire substrate-tip bias. Within the bias-driven single level model – which turned out to be successful in describing the presently analyzed *I*–*V* asymmetry (“rectification”) – current rectification is very sensitive to the values of *γ* (RR = 1 for *γ* = 0). To illustrate this point, in Fig. 7C we present curves for rectification at biases accessed in experiments and values of *γ* ranging from *γ* = –0.05 to *γ* = –0.3; where, the lowest *γ*-value corresponds to the experimental situation (*cf.*Table 1) while the latter value represents an average of the theoretical *γ*-values obtained within OVGF calculations. The message conveyed by Fig. 7C should be clear: rectifications RR ≈ 500 (see the curve for *γ* = –0.3) comparable to reasonably high values reported in the literature can be reached if improved contact engineering made it possible that the active HOMO level would feel the entire applied bias.

From the foregoing perspective, identifying and implementing appropriate electrically transparent contacts (no screening) appears to be an important problem for molecular rectification. In this vein, it is not at all surprising that significant improvements of molecular rectifiers were obtained with non-traditional approaches of contacting the SAM to electrodes. Attempting to employ platforms wherein covalent coupling between the electrode and the “active” unit (thiol in our case) on which the dominant HOMO orbital is centered is replaced by non-covalent interactions (*e.g.*, van der Waals interactions) seems to be a meaningful route to pursue in view of the recent rectification improvements achieved in this way (the weak coupling regime).^14^,^45^,^69^

## Conclusion

In this paper, we have reported results of a joint experimental–theoretical investigation of the transport properties of CP-AFM molecular junctions based on alkane monothiols (CnT) of various lengths *n* and metallic electrodes (Ag, Au, and Pt) having work functions *Φ* varying within a broad range of ∼1.4 eV. An important aspect of the present study, which enables us to propose a coherent picture of the transport, is the validation of the model of a single dominant level that is linearly shifted by the applied bias (linear Stark effect as opposed to quadratic Stark effect in atoms).

Contrary to what one expects intuitively based on the potentiometer rule, CnT junctions exhibit very modest rectification ratios (RR ≈ 1.5). By corroborating the experimental results on nanojunctions with state-of-the art *ab initio* quantum chemical calculations based on the outer valence Green's function (OVGF) method for isolated molecules, we are able to understand why this rectification is so unexpectedly weak. Namely, we have demonstrated that it is only a small fraction of the applied voltage that is responsible for the bias driven shift of the dominant level energy, which in turn is responsible for current rectification. This is a clear indication that phenomena occurring at molecule–electrode contacts are responsible for the unexpected weak rectification of CnT junctions. We believe that this finding represents an important new insight into the role played by contacts in mitigating the impact of the applied bias on the embedded molecule (possibly *via* interface states), which is an essential point to consider for improving the performance of molecular rectifiers.

## Experimental section

### Materials

1-Heptanethiol (C7T) 98%, 1-octanethiol (C8T) 98.5%, 1-nonanethiol (C9T) 99%,1-decanethiol (C10T) 99%, and 1-dodecanethiol (C12T) 98% were purchased from Sigma Aldrich. Gold nuggets (99.999% pure) were purchased from Mowrey, Inc. (St. Paul, MN). Silver pellets (99.99% pure) were purchased from Kurt J. Lesker Company. Evaporation boats and chromium evaporation rods were purchased from R. D. Mathis (Long Beach, CA). Platinum and titanium for e-beam evaporation were purchased from Kamis, Inc. (Mahopac Falls, NY). Silicon (100) wafers were obtained from WaferNet (San Jose, CA). Contact mode AFM tips (DNP-10 silicon nitride probes) were purchased from Bruker AFM Probes.

### Conducting tip and sample preparation

Contact mode AFM tips were coated with Ag, Au and Pt. Template-stripped flat metal substrates were used to grow high quality self-assembled monolayers (SAMs) for sample characterization and reproducible electrical measurements. The preparation of AFM tips and template stripped flat substrates with Ag, Au and Pt has been described previously.^70^,^71^ SAMs were formed by immersing clean template-stripped flat metal substrates in ethanol solution of molecules at a concentration of 1–2 mM for 20 h. Afterward, the samples were rinsed with ethanol and dried with flowing N_2_.

### Transport measurements

The electrical measurements were completed by mounting the substrates in the AFM and bringing the metal coated tip into contact with the SAM under ∼1 nN of applied compressive load, Fig. 1A. The voltages were applied to the tip with a Keithley model 236 electrometer operated in “DC mode”. Voltage was swept at the tip, and *I*–*V* characteristics were recorded; *V* > 0 means a positive tip (electric field pointing to the thiol/substrate, *cf.*Fig. 4). All measured *I*–*V* curves were linear at low biases and nonlinear at higher biases. The inverse slope of the linear portion of the *I*–*V* characteristics was employed to define a junction (ohmic) resistance. The low bias resistance was measured between ±0.1 V except the data of C12T, which were collected between ±0.5 V due its low conductivity, and ±1.5–2 V was applied to the tip to get the transition voltage *V*_t_.

## Theory section

### Quantum chemical calculations

The quantum chemical calculations were based on the OVGF method.^53^,^54^ For the medium-size molecular species in external electric fields considered in this study, the OVGF method represents the state-of-the-art quantum chemistry. This method was successfully applied to molecular species and sizes of interest for molecular electronics in several recent studies^72^–^74^ wherein effects of an external field were not considered. OVGF calculations were done using the implementation in the GAUSSIAN 09 suite of programs,^75^ as it allows calculations with external fields. OVGF calculations using GAUSSIAN 09 in an external electric field can be done by appropriately setting the relevant keywords. To exemplify, in the case of an external field of 8 GAUSSIAN units (1 GAUSSIAN unit = 10^–4^ a.u. = 0.05142 V nm^–1^) along the negative *y* axis, we set “FIELD = Y-8 NoSymm EPT = OVGF TRAN = FULL IOP(9/11 = 100)” in the GAUSSIAN*.com input file. Within the OVGF framework the HOMO energies (shown in Fig. 6) are estimated from the poles of the one-particle Green's function. The HOMO energy represents the lowest ionization energy with reversed sign.

## Conflicts of interest

There are no conflicts to declare.

## Supplementary Material

Supplementary information
