# Supplementary material for: Why one can expect large rectification in molecular junctions based on alkane monothiols and why rectification is so modest
Source: Chem Sci. 2018 Apr 9;9(19):4456–67. doi: 10.1039/c8sc00938d (PMC5956982; doi:10.1039/c8sc00938d)
Supplement: Supplementary file 1 [file SC-009-C8SC00938D-s001.pdf]

Electronic Supplementary Material (ESI) for Chemical Science.
This journal is © The Royal Society of Chemistry 2018

ELECTRONIC SUPPORTING INFORMATION for

Why One Can Expect Large Rectification in Molecular Junctions Based on Alkane Monothiols and Why Rectification Is So Modest

Zuoti Xie,[‡,†] Ioan Bâldea,[§,†,*] and C. Daniel Frisbie[‡,*]

[‡]*Department of Chemical Engineering and Materials Science and Department of Chemistry, University of Minnesota, Minneapolis, Minnesota 55455, United States*
[§]*Theoretische Chemie, Universität Heidelberg, INF 229, D-69120 Heidelberg, Germany*

† Equal contribution
*To whom correspondence should be addressed
E-mail: ioan.baldea@pci.uni-heidelberg.de; frisbie@umn.edu

Figure S1: Schematic representation of the potentiometer rule.

Figure S2: Geometry of alkane monothiols (CnT, n= 9 and 12) used in this study.

Figure S3: Low bias resistance of Metal-CnT-Metal junctions.

Figure S4: Semilog *I*-*V* plots of Metal-CnT-Metal junctions.

Figure S5: Transition voltages $V_{t\pm}$ of Metal-CnT-Metal junctions.

Figure S6: Interface coupling Γ of Metal-CnT-Metal junctions.

Figure S7: Histograms for the Stark effect strength γ of Metal-CnT-Metal junctions.

Figure S8: The good agreement between the individual experimental *I*-*V* curves for Metal-CnT-Metal and those obtained theoretically *via* Eq. 1.

Figure S9: LUMO energies of the alkanethiol series CnT (n=7, 8, 9, 10) as a function of the applied electric field $\mathcal{E}$ and bias V.

Potentiometer rule

The potentiometer rule assumes that screening effects within the junction are altogether ineffective, and that the contacts (*i.e.*, the electrode-molecule interfaces) do in no way affect the electric potential. Then, the potential profile $V(z)$ across the junction is simply that of a region characterized by a constant electric field, varying linearly (cf. Figure S1) along the junction

$$V(z) = -V\frac{z}{d} \quad \text{(S1)}$$

Here the coordinate z

$$-d/2<z<d/2 \quad \text{(S2)}$$

is the measured from the center, and $V \equiv V_t - V_s = V/2-(-V/2)$ is the difference between the potentials $V_{t,s}$ of the tip *(t)* and substrate *(s)*. Within this picture, the energy shift of the nearly point-like MO strongly localized around $z=z_{MO}$ caused by the applied bias V can be expressed as

$$\delta\varepsilon_0 \equiv \varepsilon_0(V)-\varepsilon_0(V=0)=-eV(z)= e\frac{z_{MO}}{d}V \quad \text{(S3)}$$

Eqs. 2 and S3 indicate a linear dependence on z_{MO} of the asymmetry parameter γ within the potentiometer rule framework

$$\gamma= \frac{z_{MO}}{d} \quad \text{(S4)}$$

That is, the closer to an electrode, the larger is the magnitude $|\gamma|$ of the asymmetry parameter, whose maximum value, corresponding to $z_{MO}=\pm d/2$ (*cf.* eq. S2), is reached in the case of an MO located very close to the molecule-electrode interface. According to eq S4 the maximum value is equal to $|\gamma_{max}|=1/2$.

The idea underlying Figure S1 is based on the above eq. S3, which visualizes the physical content of the potentiometer rule.

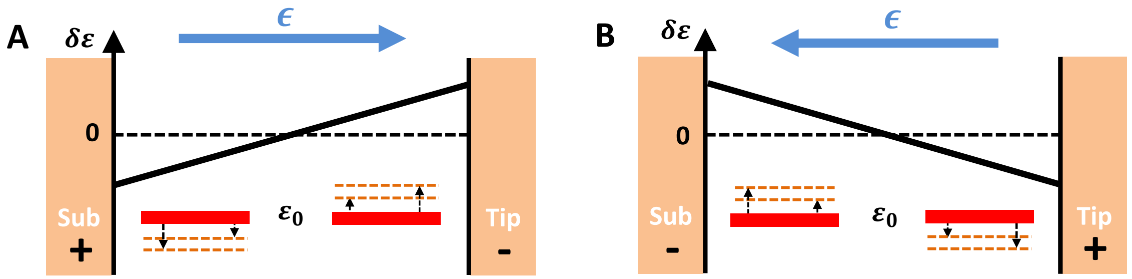

Figure S1. Schematic representation of the potentiometer rule based on eq. S3. The MO energy offset $\varepsilon_0(V)$ is shifted by the bias V that depends on the MO location. In the absence of screening and potential drops at contacts, the magnitude of the bias-driven energy shift is larger for an MO located closer to a molecular end/electrode. (A) Negative bias on the tip, V<0, (B) positive bias on the tip, V>0.

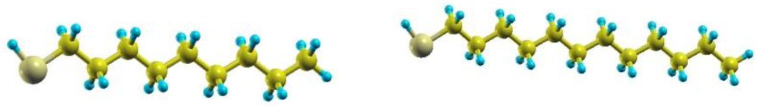

Figure S2. Examples of alkane monothiol molecules (CnT) used in the present study: C9T and C12T.

Charge Transport in Low Bias Range. Our results for the low bias resistance are presented in Figure S3. In discussing these results, we will separately consider the impact of the molecular length (n) and the contact (metal) nature. For homologous molecular series, the two aforementioned effects can be conveniently disentangled by expressing the low bias resistance $R = R_n$ analyzed as follows[1]

$$R_n = R_c \exp(\beta n L_0) \qquad \text{(S5)}$$

Here R_c is the effective contact resistance, β is the tunneling decay parameter, $L_0 \approx 1.2$ Å is the repeat unit length, and n is the number of repeating units. The exponential length

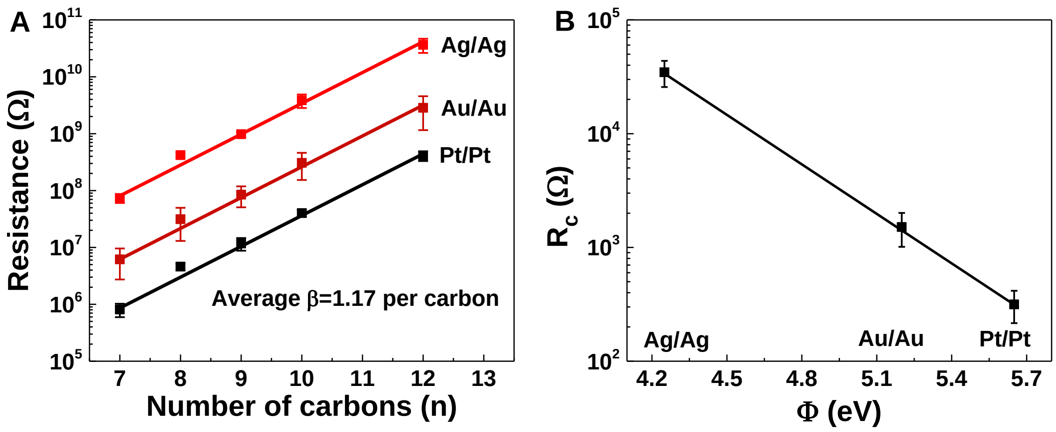

Figure S3. (A) Semilog plot of low bias resistance of CnT (n=7, 8, 9, 10, 12) *versus* repeat units n. (B) Semilog plot of the contact resistance R_C *versus* the work functions of the bare electrodes.

dependence shown in the equation represents a general feature of off-resonant tunneling. From the slope of the semilogarithmic plot of $R = R_n$ *versus* n, one can determine the tunneling attenuation factor β, while its intercept at $n = 0$ gives the effective contact resistance R_c. Low bias resistances of CnT as well as the values of β and R_c for the various types of junctions are shown in Table 1 of the main text.

Tunneling Attenuation Factor. Figure S3A displays a semilogarithmic plot of resistance versus number of carbons for CnT junctions. Resistances were calculated from the average of about 200 *I-V* traces within ±0.3V. The exponential length dependence of low bias resistance of CnT junctions (Table 1 and Figure S3A) are well explained within the off-resonant tunneling picture underlying Eq. S5. The average tunneling attenuation factor β value determined from the slopes of the semilogarithmic plots of Figure S3A (1.17 per carbon or 0.98 $Å^{-1}$) agree with previous reports.[1–9] Consistent with our previous

observation,[9] β values of CnT junctions are independent of the type of the metallic electrodes while the contact resistances decrease by a factor of 110 depending upon the electrodes in the order of $R_{C\text{-}Ag} > R_{C\text{-}Au} > R_{C\text{-}Pt}$. Since the β value is related to the energy offset of the dominant MO, we primarily ascribe its independence of metal to Fermi level pinning, an effect we have also observed previously in CP-AFM junctions based on aromatic molecules.[10,11]

Contact Resistance versus Electrode Work Function. The contact resistance R_c determined from the zero-length intercept clearly indicates the important role of the type of electrodes. As visible in Figure S3B, the bare electrode work function Φ has a dramatic effect on the contact resistance R_c. For the electrodes studied (Ag, Au, and Pt) Φ varies by 1.4 eV, and this manifests itself in a (contact) resistance decreasing by a factor of 110. As noted earlier,[10,11] the opposite variations in Φ and R reveal a hole (p-type HOMO-mediated) conduction. The dramatic decrease in R_c with Φ for the presently considered alkane monothiol junctions is comparable to that of our previous studies on CP-AFM junctions based on alkane dithiols.[9]

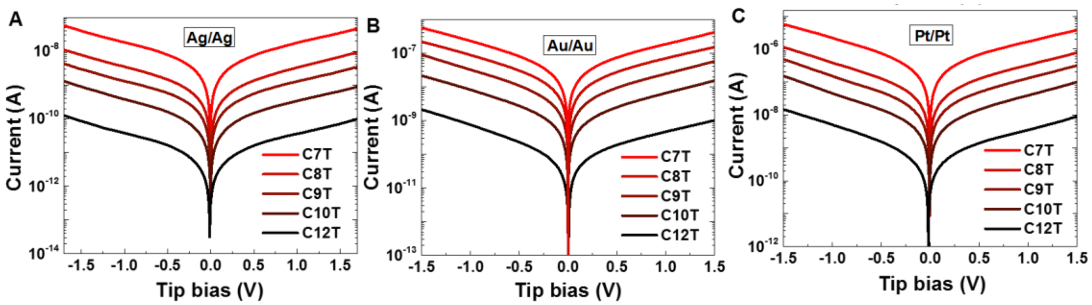

Figure S4. Representative semilog *I-V* plots obtained by averaging ~50 *I-V* traces obtained from homo-metal M-CnT-M (n=7, 8, 9, 10 and 12) CP-AFM junctions (A) M=Ag, (B) M=Au, and (C) M=Pt.

Determination of the Model Parameters.

HOMO-Fermi energy offset ε_h. In line with the philosophy underlying TVS,[12] we use the $V_{t\pm}$ values (*cf.* Table 1, Figure S5) extracted from *I-V* measurements to estimate the energy offset ε_h=-ε_0 *via* eq. 4 deduced earlier[13] for the asymmetric single level model.

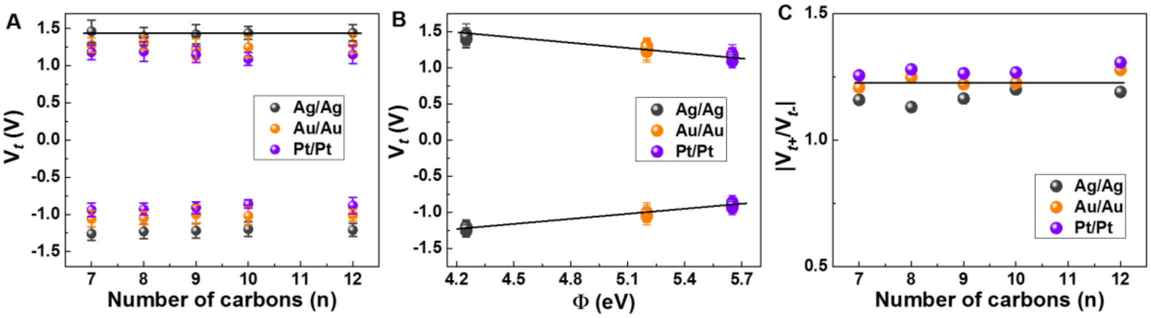

Figure S5. Transition voltages $V_{t\pm}$ of M-CnT-M junctions as a function of (A) number of the repeat units and (B) bare electrode work function. (C) The ratios of V_{t+}/V_{t-} of M-CnT-M junctions. (n=7, 8, 9, 10, 12; M=Ag, Au, Pt)

The calculated energy offsets ε_h of CnT junctions with different metal contacts are listed in Table 1. As shown in Figure 3A, ε_h are independent of the length of the molecule (*n*). This agrees with our quantum chemical calculations (not shown here) indicating that the HOMO energies of the isolated alkane thiols are practically independent of the molecular size n. On the other hand, the HOMO energy offset slightly decreases with increasing work function of the contact metals, Figure 3B. Specifically, Table 1 shows that the ε_h values change only within ~0.3 eV over a 1.4 eV change in electrode work function. This is an indication of strong Fermi level pinning effect of the HOMO for these junctions. This behavior is in line with our previous findings by UPS and the single level model on aromatic molecules.[10,11] Since the tunneling attenuation factor $\beta \propto (|\varepsilon_0|)^{1/2}$,[9,14,15] the small

variation in $|\varepsilon_0|$ induced by different metallic electrodes is likely responsible for the nearly unchanged β values.

<u>Number (*N*) of Alkane thiol in the junction.</u> To estimate the value of the interface coupling strength via eq. 3, the number of the molecule in the junction is needed. SAM-coverages of alkane thiols, amounting to 3.5 molecules/nm^2, are known from our recent Rutherford backscattering and nuclear reaction analysis studies[16]. To determine contact areas we employed established contact mechanics methods[17]. Calculations based on the Maugis-Dugdale (MD) model of contact mechanics[18] yield for CnT values of ~20 nm^2 for contact areas at 1 nN. With the values of contact area and molecular coverage in hand, we estimated the molecule number *N* in the junctions, which was found to be *N*≈70.

<u>Contact Coupling Strength *Γ*.</u> With the energy offset ε_h and the number of the molecule (N)

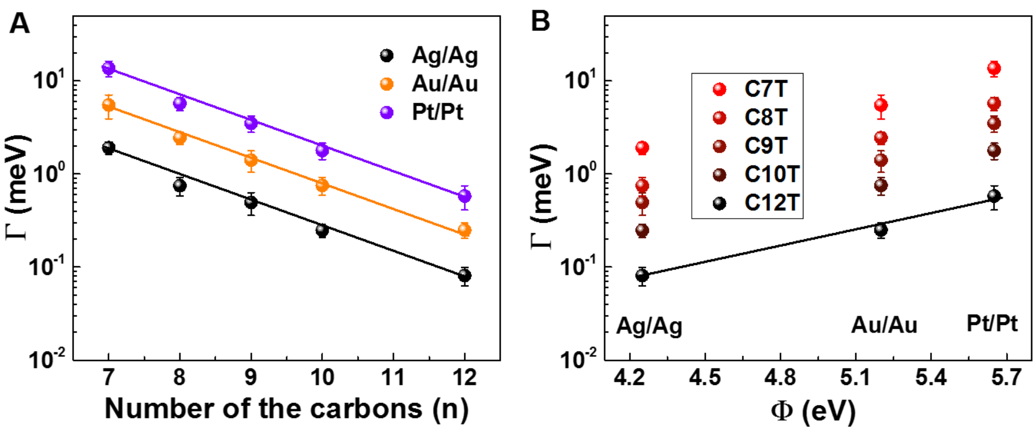

Figure S6. Interface coupling Γ of M-CnT-M junctions (M=Ag, Au, Pt) as a function of (A) molecular length and (B) bare electrodes' work function. The lines represent linear fits.

in hand, in addition to the value of the low conductance *G*(≡*1/R*), we calculate the average molecule electrode coupling *Γ* using eq. 3. As visualized in Figures S6A and S6B, *Γ* falls off exponentially with *n* over one order of magnitude for each type of metal contact and

increases exponentially with increasing of Φ. The length independence of $|\varepsilon_0|$ as well as the pinning effect resulting in small changes in $|\varepsilon_0|$ despite the large span of work function of different contact electrodes suggest that the behavior of $|\varepsilon_0|$ cannot be responsible for the dramatic increase of R with *n* and the decrease of R_c (and *R*) with increasing *Φ*. Rather, in accord with Figure S6, the strong dependence of R on *n* and *Φ* and of R_c on *Φ* is primarily determined by the changes in *Γ*.

Stark Effect Strength γ. Values of γ for individual *I-V* traces were calculated by means of eq. 5 of the main text. γ-histograms are shown in Figure S7; average values and statistical deviations are presented in Table 1 in the main text.

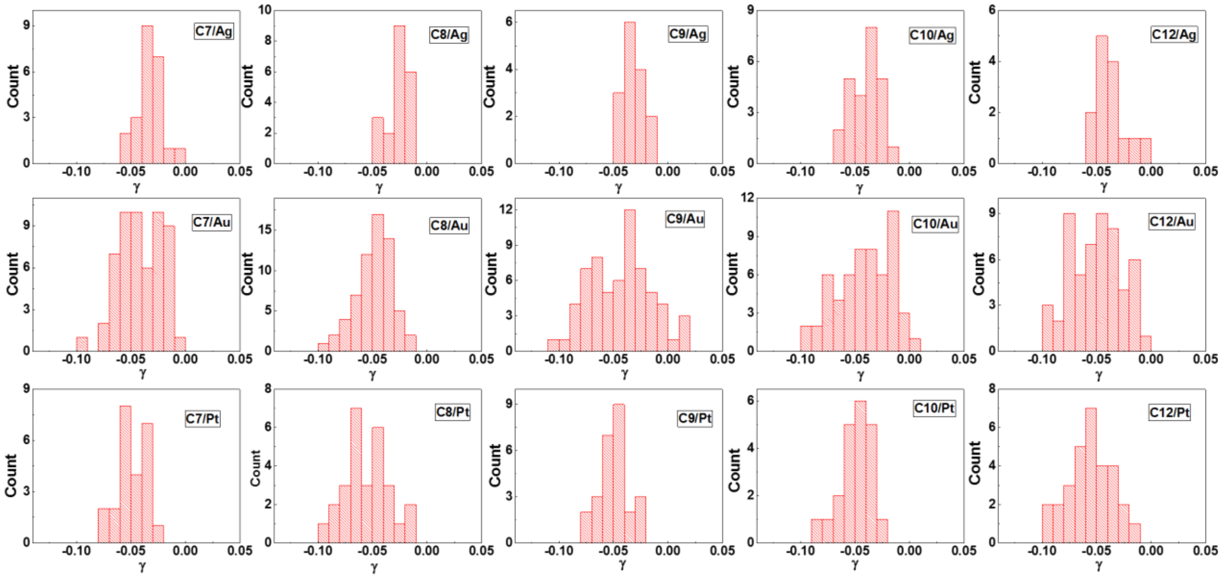

Figure S7. Histograms for the Stark effect strength γ of M-CnT-M junctions. (n= 7, 8, 9, 10, 12 and M= Ag, Au and Pt)

Simulation of Full I-V Curves Using the Asymmetric Single Level Model. To more convincingly illustrate the accuracy of the theoretical description based on the single level

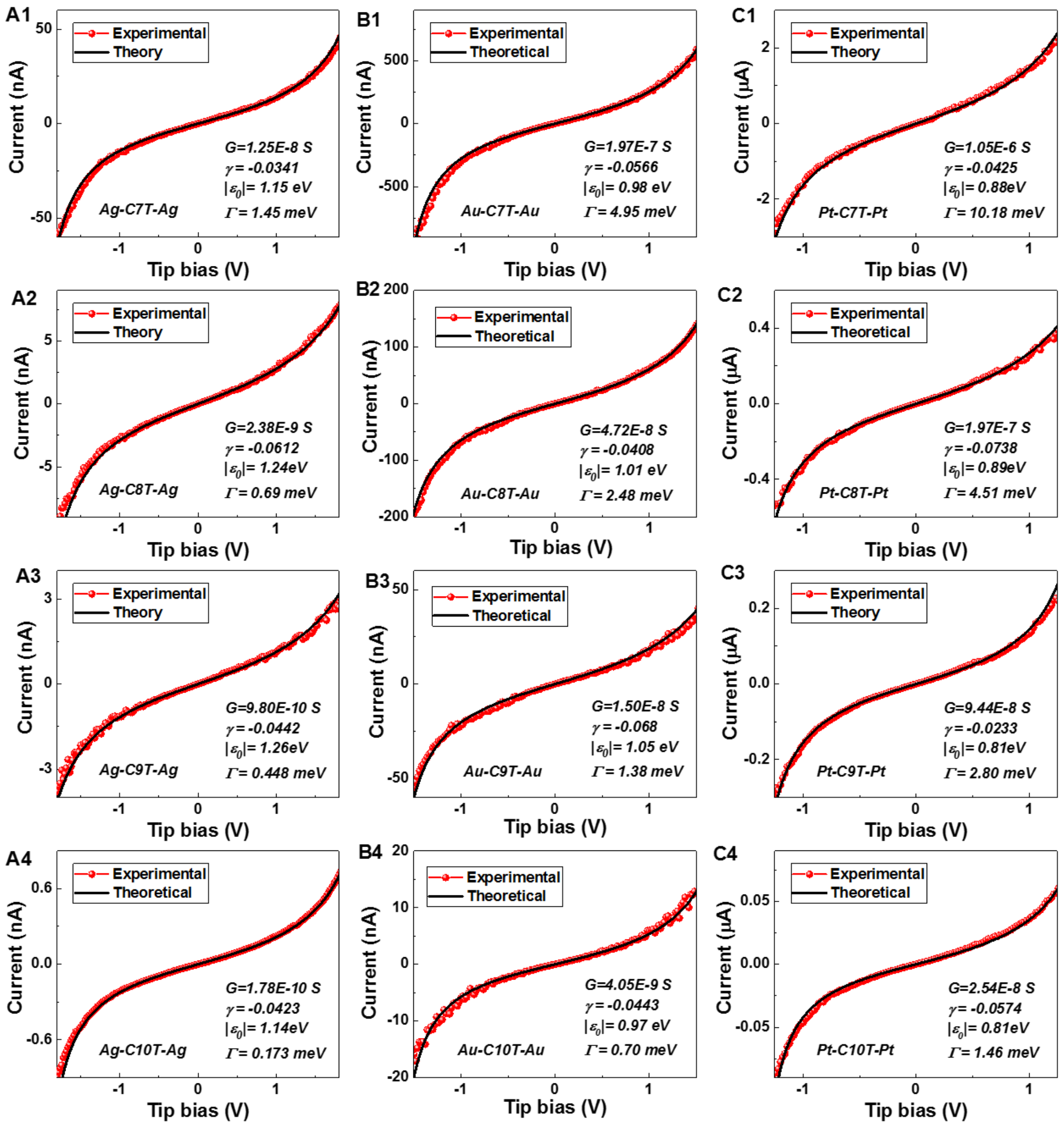

Figure S8. The good agreement between the individual experimental *I-V* curves (red) and those obtained theoretically *via* eq. 1 (black) is illustrated here for (A1-A4) Ag-CnT-Ag, (B1-B4) Au-CnT-Au and (C1-C4) Pt-CnT-Pt junctions. (n = 7, 8, 9, 10) Model parameter values are indicated in the legends. Bias fitting ranges (X axis) are 1.8 V, 1.5 V and 1.25 V for Ag, Au and Pt contacts respectively. Currents on Y axis are in nA for Ag and Au contacts, and in μA for Pt contacts.

model underlying eq. 1 and 2 of the main text, in addition to the curves depicted in Figure 5 of the main text, further examples are presented in Figure S8.

OVGF results of LUMO energies of the alkanethiol *vs.* applied electric field ϵ / bias V_m between the molecular ends. OVGF calculations of the LUMO energy in external field provide us with an extra argument against LUMO-mediated conduction. A rectification like that we observed in experiment (i.e., $I(+V)<|I(-V)|$ for V>0) would require a LUMO-energy offset ($\varepsilon(V)\equiv E_{LUMO}(V)-E_F$) larger at a positive bias ($V>0$) than that at the corresponding negative bias ($-V$); that is, $\varepsilon_0(V)\equiv E_{LUMO}(V)-E_F> \varepsilon_0(-V)\equiv E_{LUMO}(-V)-E_F >0$, or, alternatively

$$E_{LUMO}(V)> E_{LUMO}(-V) \text{ for } V>0 \qquad \text{(S6)}$$

In Figure S9A, we present results for the LUMO energy for alkanethiol molecules in external field. They clearly invalidate the requirement imposed by eq. S6; the bias-driven LUMO energy shifts (Figure S9B) are almost independent of the bias polarity.

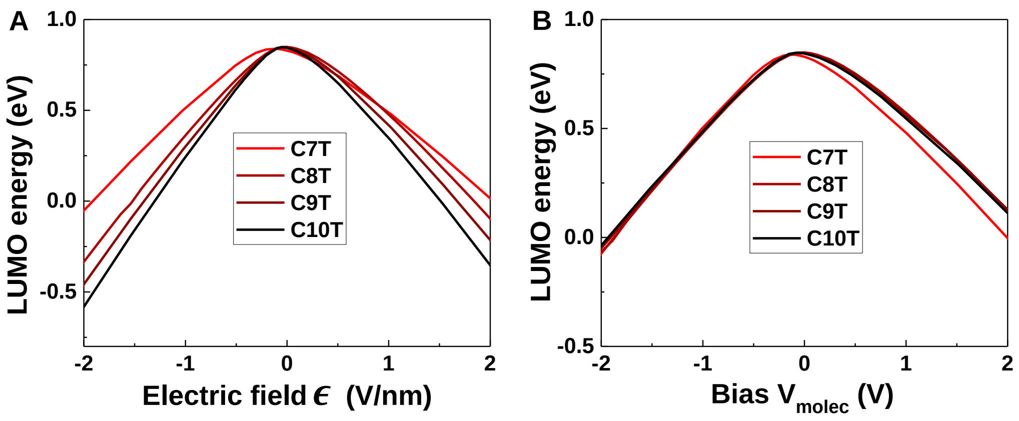

Figure S9. LUMO energies of the alkanethiol series CnT (n=7, 8, 9, 10) as a function of the (A) applied electric field ϵ and (B) bias V_m between the molecular ends. Results of quantum chemical computations based on the OVGF.

Reference:

1 D. J. Wold and C. D. Frisbie, *J. Am. Chem. Soc.*, 2000, **122**, 2970–2971.

2 K. Slowinski, R. V Chamberlain, C. J. Miller and M. Majda, *J. Am. Chem. Soc.*, 1997, **119**, 11910–11919.

3 J. F. Smalley, S. W. Feldberg, C. E. D. Chidsey, M. R. Linford, M. D. Newton and Y.-P. Liu, *J. Phys. Chem.*, 1995, **99**, 13141–13149.

4 L. A. Bumm, J. J. Arnold, T. D. Dunbar, D. L. Allara and P. S. Weiss, *J. Phys. Chem. B*, 1999, **103**, 8122–8127.

5 D. J. Wold and C. D. Frisbie, *J. Am. Chem. Soc.*, 2001, **123**, 5549–5556.

6 C. S. S. Sangeeth, A. Wan and C. A. Nijhuis, *J. Am. Chem. Soc.*, 2014, **136**, 11134–11144.

7 M. M. Thuo, W. F. Reus, F. C. Simeone, C. Kim, M. D. Schulz, H. J. Yoon and G. M. Whitesides, *J. Am. Chem. Soc*, 2012, **134**, 10876–10884.

8 H. Song, Y. Kim, H. Jeong, M. A. Reed and T. Lee, *J. Phys. Chem. C*, 2010, **114**, 20431–20435.

9 V. B. Engelkes, J. M. Beebe and C. D. Frisbie, *J. Am. Chem. Soc.*, 2004, **126**, 14287–14296.

10 B. Kim, S. H. Choi, X.-Y. Zhu and C. D. Frisbie, *J. Am. Chem. Soc.*, 2011, **133**, 19864–19877.

11 Z. Xie, I. Bâldea, C. E. Smith, Y. Wu and C. D. Frisbie, *ACS Nano*, 2015, **9**, 8022–8036.

12 J. M. Beebe, B. Kim, J. W. Gadzuk, C. D. Frisbie and J. G. Kushmerick, *Phys. Rev. Lett.*, 2006, **97**, 26801.

13 I. Bâldea, *Phys. Rev. B*, 2012, **85**, 35442.

14 A. Salomon, T. Boecking, O. Seitz, T. Markus, F. Amy, C. Chan, W. Zhao, D. Cahen and A. Kahn, *Adv. Mater.*, 2007, **19**, 445–450.

15 A. Salomon, T. Boecking, C. K. Chan, F. Amy, O. Girshevitz, D. Cahen and A. Kahn, *Phys. Rev. Lett.*, 2005, **95**, 266807.

16 A. T. Demissie, G. Haugstad and C. D. Frisbie, *J. Phys. Chem. Lett.*, 2016, **7**, 3477–3481.

17 G. Haugstad, *Atomic Force Microscopy*, John Wiley&Sons, Inc, Hoboken, New Jersey, 2012.

18 Z. Xie, I. Bâldea, A. T. Demissie, C. E. Smith, Y. Wu, G. Haugstad and C. D. Frisbie, *J. Am. Chem. Soc.*, 2017, **139**, 5696–5699.
